# The Study of Dynamical Potentials of Highly Excited Vibrational States of HOBr

**DOI:** 10.3390/ijms14035250

**Published:** 2013-03-05

**Authors:** Aixing Wang, Lifeng Sun, Chao Fang, Yibao Liu

**Affiliations:** 1 Engineering Research Center of Nuclear Technology Application (East China Institute of Technology), Ministry of Education, Nanchang 330013, China; E-Mails: xingxing_fz@sina.com (A.W.); liuyb01@mails.tsinghua.edu.cn (Y.L.); 2 College of Science, East China Institute of Technology, Nanchang 330013, China; 3 Institute of Nuclear and New Energy Technology, Tsinghua University, Beijing 100084, China; E-Mail: lfsun@tsinghua.edu.cn

**Keywords:** HOBr, highly excited vibrational state, geometrical shape of dynamical potential, phase space trajectory

## Abstract

The vibrational nonlinear dynamics of HOBr in the bending and O–Br stretching coordinates with anharmonicity and Fermi 2:1 coupling are studied with dynamical potentials in this article. The result shows that the H–O stretching vibration mode has significantly different effects on the coupling between the O–Br stretching mode and the H–O–Br bending mode under different Polyad numbers. The dynamical potentials and the corresponding phase space trajectories are obtained when the Polyad number is 27, for instance, and the fixed points in the dynamical potentials of HOBr are shown to govern the various quantal environments in which the vibrational states lie. Furthermore, it is also found that the quantal environments could be identified by the numerical values of action integrals, which is consistent with former research.

## 1. Introduction

Atmospheric pollution has caused widespread concern because it has led to not only a lot of diseases but also serious damage to the ecological balance. HOBr is a very unstable oxidizing substance in the atmosphere. Radical generation of Bromine (Br) is caused by the O–Br bond breaking in highly excited vibrations of HOBr molecules. It is known that photolysis of the refrigerator refrigerant Freon produces bromine (Br), which leads to the destruction of atmospheric O_3_[1]. It has been shown that OH radicals and halogen atoms are the main sources of the formation of photochemical smog, and, therefore, the study of the dynamical properties of HOBr molecules in highly excited vibration has attracted a lot of attention.

The dynamics of bending and OBr stretching vibration of HOBr molecules have already been fully studied, and the main research methods are the first principle calculations or semi-classical methods [2–9]. Compared with the first-principle calculation, the semi-classical method can provide a more intuitive physical image and avoid the tedious calculations. Fruitful achievements of the research of highly excited vibration have been obtained by semi-classical methods, and some important conclusions provide a way to understand molecular dynamics characteristics [6,7]. In recent years, a new semi-classical method “dynamics potential” [5–7] has been proposed and applied to study highly excited vibrational molecular states. Applications of a dynamical potential phase and space analysis could both verify the conclusion of the first-principle method and give detailed physical pictures, including molecular isomerization [10], chaotic dynamics, dissociation dynamics [11–13] and other information.

In this work, the dynamic potentials of highly excited vibrational states of HOBr in bending and of O–Br stretching coordinates with anharmonic resonance and Fermi coupling will be shown. The effect of the H–O stretching vibration mode on the O–Br stretching mode and the H–O–Br bending mode under different Polyad numbers will be investigated and the dynamical potentials, including the corresponding phase space trajectories will also be studied, which is helpful to understand the dynamics of highly excited vibrational states.

## 2. The Semi-Classical Hamiltonian of the HOBr System

The dynamical properties of highly excited vibrational states of HOBr in the energy region of 5 × 10^3^–2.5 × 10^4^ cm^−1^ are abundant and are the focus of this work [2,9,13]. The HOBr vibration Hamiltonian in the energy region and its corresponding coefficient can be obtained [2,13] as follows:

(1) $$\begin{array}{l} H=\omega_{1}(n_{1}+\frac{1}{2})+\omega_{2}(n_{2}+\frac{1}{2})+\omega_{3}(n_{3}+\frac{1}{2})\\ +x_{11}{\left(n_{1}+\frac{1}{2}\right)}^{2}+x_{12}(n_{1}+\frac{1}{2})(n_{2}+\frac{1}{2})+x_{13}(n_{1}+\frac{1}{2})(n_{3}+\frac{1}{2})\\ +x_{22}{\left(n_{2}+\frac{1}{2}\right)}^{2}+x_{23}(n_{2}+\frac{1}{2})(n_{3}+\frac{1}{2})+x_{33}{\left(n_{3}+\frac{1}{2}\right)}^{2}\\ +y_{113}{\left(n_{1}+\frac{1}{2}\right)}^{2}(n_{3}+\frac{1}{2})+y_{122}(n_{1}+\frac{1}{2}){\left(n_{2}+\frac{1}{2}\right)}^{2}+y_{133}(n_{1}+\frac{1}{2}){\left(n_{3}+\frac{1}{2}\right)}^{2}\\ +y_{223}{\left(n_{2}+\frac{1}{2}\right)}^{2}(n_{3}+\frac{1}{2})+z_{1133}{\left(n_{1}+\frac{1}{2}\right)}^{2}{\left(n_{3}+\frac{1}{2}\right)}^{2}+z_{1333}(n_{1}+\frac{1}{2}){\left(n_{3}+\frac{1}{2}\right)}^{3}\\ +z_{2333}(n_{2}+\frac{1}{2}){\left(n_{3}+\frac{1}{2}\right)}^{3}+z_{3333}{\left(n_{3}+\frac{1}{2}\right)}^{4}+z_{11112}{\left(n_{1}+\frac{1}{2}\right)}^{4}(n_{2}+\frac{1}{2})\\ +z_{11122}{\left(n_{1}+\frac{1}{2}\right)}^{3}{\left(n_{2}+\frac{1}{2}\right)}^{2}+z_{11222}{\left(n_{1}+\frac{1}{2}\right)}^{2}{\left(n_{2}+\frac{1}{2}\right)}^{3}+z_{11223}{\left(n_{1}+\frac{1}{2}\right)}^{2}{\left(n_{2}+\frac{1}{2}\right)}^{2}(n_{3}+\frac{1}{2})\\ +z_{12222}(n_{1}+\frac{1}{2}){\left(n_{2}+\frac{1}{2}\right)}^{4}+z_{22233}{\left(n_{2}+\frac{1}{2}\right)}^{3}{\left(n_{3}+\frac{1}{2}\right)}^{2}+z_{22333}{\left(n_{2}+\frac{1}{2}\right)}^{2}{\left(n_{3}+\frac{1}{2}\right)}^{3}\\ +[{\text{k}}_{1}(n_{1}+\frac{1}{2})+{\text{k}}_{2}{\text{n}}_{2}+{\text{k}}_{3}({\text{n}}_{3}+\frac{3}{2})+{\text{k}}_{22}{\text{n}}_{2}^{2}]({\text{a}}_{2}^{+}{\text{a}}_{3}^{2}+{\text{a}}_{3}^{+2}{\text{a}}_{2}) \end{array}$$

The corresponding coefficients are shown in Table 1, where the Subscripts 1, 2, and 3, respectively, correspond to the H–O stretching vibration mode, the mode of the bending vibration between the angle of H–O and O–Br, and the O–Br bond stretching vibration mode. The values *a^+^* and *a* are the creation and the destruction operators, which represent the increase or decrease of the vibration mode corresponding to the quantum number. The value *n* indicates the quantum number of the vibration modes (for convenience, hereinafter we also use *n* to denote the corresponding vibration modes, which will be indicated with a *q* in the position coordinates and the momentum coordinates will be indicated with a *p*). The value *ω* is the corresponding harmonic vibration coefficient, while *x*, *y*, *z* denote the nonlinear coupling coefficients among the different modes. Strong 2:1 Fermi resonance coupling exists between the *n*_2_ and *n*_3_ mode in HOBr and is observed in reference [2,3], and the strength of this resonance is related to the quantum numbers of the three modes. Based on the above-mentioned reasons, k represents the Fermi resonance strength coefficient in Hamiltonian between the bending vibration and O–Br stretching vibration.

Besides the conserved quantities of the *n*_1_, considering the 2:1 Fermi resonance, 2*n*_2_ + *n*_3_ is also a conserved quantity as a whole, which is called the Polyad number (P number). It is known that Equation (1) is available to study the dynamical properties of highly excited vibrational states of HOBr in the region of *n*_1_ ≤ 7, *P* ≤ 31 [9].

It is also easy to semi-classify the second quantization Hamiltonian (1), which is useful in the further analysis. The coset space SU(2)/U(1) [14] can be used as the representing space of HOBr’s Hamiltonian, so it can be rewritten using the bending coordinates (*q*_2_, *p*_2_) as follows:

(2) $$\begin{array}{l} H(n_{1},q_{2},p_{2},P)=\omega_{1}(n_{1}+\frac{1}{2})+\omega_{2}(\frac{p_{2}^{2}+q_{2}^{2}}{2}+\frac{1}{2})+\omega_{3}(P-(p_{2}^{2}+q_{2}^{2})+\frac{1}{2})+X_{11}{\left(n_{1}+\frac{1}{2}\right)}^{2}\\ +X_{12}(n_{1}+\frac{1}{2})(\frac{p_{2}^{2}+q_{2}^{2}}{2}+\frac{1}{2})+X_{13}(n_{1}+\frac{1}{2})(P-(p_{2}^{2}+q_{2}^{2})+\frac{1}{2})+X_{22}{\left(\frac{p_{2}^{2}+q_{2}^{2}}{2}+\frac{1}{2}\right)}^{2}\\ +X_{23}(\frac{p_{2}^{2}+q_{2}^{2}}{2}+\frac{1}{2})(P-(p_{2}^{2}+q_{2}^{2})+\frac{1}{2})+X_{33}{\left(P-(p_{2}^{2}+q_{2}^{2})+\frac{1}{2}\right)}^{2}\\ +y_{113}{\left(n_{1}+\frac{1}{2}\right)}^{2}(P-(p_{2}^{2}+q_{2}^{2})+\frac{1}{2})+y_{122}(n_{1}+\frac{1}{2}){\left(\frac{p_{2}^{2}+q_{2}^{2}}{2}+\frac{1}{2}\right)}^{2}\\ +y_{113}(n_{1}+\frac{1}{2}){\left(P-(p_{2}^{2}+q_{2}^{2})+\frac{1}{2}\right)}^{2}+y_{223}{\left(\frac{p_{2}^{2}+q_{2}^{2}}{2}+\frac{1}{2}\right)}^{2}(P-(p_{2}^{2}+q_{2}^{2})+\frac{1}{2})\\ +z_{1133}{\left(n_{1}+\frac{1}{2}\right)}^{2}{\left(P-(p_{2}^{2}+q_{2}^{2})+\frac{1}{2}\right)}^{2}+z_{1333}(n_{1}+\frac{1}{2}){\left(P-p_{2}^{2}+q_{2}^{2}+\frac{1}{2}\right)}^{3}\\ +z_{2333}(\frac{p_{2}^{2}+q_{2}^{2}}{2}+\frac{1}{2}){\left(P-(p_{2}^{2}+q_{2}^{2})+\frac{1}{2}\right)}^{3}+z_{3333}{\left(P-(p_{2}^{2}+q_{2}^{2}+\frac{1}{2}\right)}^{4}\\ +z_{11112}{\left(n_{1}+\frac{1}{2}\right)}^{4}(\frac{p_{2}^{2}+q_{2}^{2}}{2}+\frac{1}{2})+z_{11122}{\left(n_{1}+\frac{1}{2}\right)}^{3}{\left(\frac{p_{2}^{2}+q_{2}^{2}}{2}+\frac{1}{2}\right)}^{2}\\ +z_{11222}{\left(n_{1}+\frac{1}{2}\right)}^{2}{\left(\frac{p_{2}^{2}+q_{2}^{2}}{2}+\frac{1}{2}\right)}^{3}+z_{11223}{\left(n_{1}+\frac{1}{2}\right)}^{2}{\left(\frac{p_{2}^{2}+q_{2}^{2}}{2}+\frac{1}{2}\right)}^{2}(P-(p_{2}^{2}+q_{2}^{2})+\frac{1}{2})\\ +z_{12222}(n_{1}+\frac{1}{2}){\left(\frac{p_{2}^{2}+q_{2}^{2}}{2}+\frac{1}{2}\right)}^{4}+z_{22233}{\left(\frac{p_{2}^{2}+q_{2}^{2}}{2}+\frac{1}{2}\right)}^{3}{\left(P-(p_{2}^{2}+q_{2}^{2})+\frac{1}{2}\right)}^{2}\\ +z_{22333}{\left(\frac{p_{2}^{2}+q_{2}^{2}}{2}+\frac{1}{2}\right)}^{2}{\left(P-(p_{2}^{2}+q_{2}^{2})+\frac{1}{2}\right)}^{3}\\ +[{\text{k}}_{1}(n_{1}+\frac{1}{2})+{\text{k}}_{2}(\frac{p_{2}^{2}+q_{2}^{2}}{2})+{\text{k}}_{3}(P-(p_{2}^{2}+q_{2}^{2})+\frac{3}{2})+{\text{k}}_{22}{\left(\frac{p_{2}^{2}+q_{2}^{2}}{2}\right)}^{2}]\sqrt{2}(P-(p_{2}^{2}+q_{2}^{2}))q_{2} \end{array}$$

With the coordinates (*q*_3_, *p*_3_), the Hamiltonian can be written as:

(3) $$\begin{array}{l} H(n_{1},q_{3},p_{3},P)=\omega_{1}(n_{1}+\frac{1}{2})+\omega_{2}(\frac{P}{2}-\frac{p_{3}^{2}+q_{3}^{2}}{4}+\frac{1}{2})+\omega_{3}(\frac{p_{3}^{2}+q_{3}^{2}}{2}+\frac{1}{2})+X_{11}{\left(n_{1}+\frac{1}{2}\right)}^{2}\\ +X_{12}(n_{1}+\frac{1}{2})(\frac{P}{2}-\frac{p_{3}^{2}+q_{3}^{2}}{4}+\frac{1}{2})+X_{13}(n_{1}+\frac{1}{2})(\frac{p_{3}^{2}+q_{3}^{2}}{2}+\frac{1}{2})+X_{22}{\left(\frac{P}{2}-\frac{p_{3}^{2}+q_{3}^{2}}{4}+\frac{1}{2}\right)}^{2}\\ +X_{23}(\frac{P}{2}-\frac{p_{3}^{2}+q_{3}^{2}}{4}+\frac{1}{2})(\frac{p_{3}^{2}+q_{3}^{2}}{2}+\frac{1}{2})+X_{33}{\left(\frac{p_{3}^{2}+q_{3}^{2}}{2}+\frac{1}{2}\right)}^{2}\\ +y_{113}{\left(n_{1}+\frac{1}{2}\right)}^{2}(\frac{p_{3}^{2}+q_{3}^{2}}{2}+\frac{1}{2})+y_{122}(n_{1}+\frac{1}{2}){\left(\frac{P}{2}-\frac{p_{3}^{2}+q_{3}^{2}}{4}+\frac{1}{2}\right)}^{2}\\ +y_{133}(n_{1}+\frac{1}{2}){\left(\frac{p_{3}^{2}+q_{3}^{2}}{2}+\frac{1}{2}\right)}^{2}+y_{223}{\left(\frac{P}{2}-\frac{p_{3}^{2}+q_{3}^{2}}{4}+\frac{1}{2}\right)}^{2}(\frac{p_{3}^{2}+q_{3}^{2}}{2}+\frac{1}{2})\\ +z_{1133}{\left(n_{1}+\frac{1}{2}\right)}^{2}{\left(\frac{p_{3}^{2}+q_{3}^{2}}{2}+\frac{1}{2}\right)}^{2}+z_{1333}(n_{1}+\frac{1}{2}){\left(\frac{p_{3}^{2}+q_{3}^{2}}{2}+\frac{1}{2}\right)}^{3}\\ +z_{2333}(\frac{P}{2}-\frac{p_{3}^{2}+q_{3}^{2}}{4}+\frac{1}{2}){\left(\frac{p_{3}^{2}+q_{3}^{2}}{2}+\frac{1}{2}\right)}^{3}+z_{3333}{\left(\frac{p_{3}^{2}+q_{3}^{2}}{2}+\frac{1}{2}\right)}^{4}\\ +z_{11112}{\left(n_{1}+\frac{1}{2}\right)}^{4}(\frac{P}{2}-\frac{p_{3}^{2}+q_{3}^{2}}{4}+\frac{1}{2})+z_{11122}{\left(n_{1}+\frac{1}{2}\right)}^{3}{\left(\frac{P}{2}-\frac{p_{3}^{2}+q_{3}^{2}}{4}+\frac{1}{2}\right)}^{2}\\ +Z_{11222}{\left(n_{1}+\frac{1}{2}\right)}^{2}{\left(\frac{P}{2}-\frac{p_{3}^{2}+q_{3}^{2}}{4}+\frac{1}{2}\right)}^{3}+z_{11223}{\left(n_{1}+\frac{1}{2}\right)}^{2}{\left(\frac{P}{2}-\frac{p_{3}^{2}+q_{3}^{2}}{4}+\frac{1}{2}\right)}^{2}(\frac{p_{3}^{2}+q_{3}^{2}}{2}+\frac{1}{2})\\ +z_{12222}(n_{1}+\frac{1}{2}){\left(\frac{P}{2}-\frac{p_{3}^{2}+q_{3}^{2}}{4}+\frac{1}{2}\right)}^{4}+z_{22233}{\left(\frac{P}{2}-\frac{p_{3}^{2}+q_{3}^{2}}{4}+\frac{1}{2}\right)}^{3}{\left(\frac{p_{3}^{2}+q_{3}^{2}}{2}+\frac{1}{2}\right)}^{2}\\ +z_{22333}{\left(\frac{P}{2}-\frac{p_{3}^{2}+q_{3}^{2}}{4}+\frac{1}{2}\right)}^{2}{\left(\frac{p_{3}^{2}+q_{3}^{2}}{2}+\frac{1}{2}\right)}^{3}\\ +[{\text{k}}_{1}(n_{1}+\frac{1}{2})+{\text{k}}_{2}(\frac{P}{2}-\frac{p_{3}^{2}+q_{3}^{2}}{4})+{\text{k}}_{3}(\frac{p_{3}^{2}+q_{3}^{2}}{2}+\frac{3}{2})+{\text{k}}_{22}{\left(\frac{P}{2}-\frac{p_{3}^{2}+q_{3}^{2}}{4}\right)}^{2}]\sqrt{\frac{P}{2}-\frac{p_{3}^{2}+q_{3}^{2}}{4}}(q_{3}^{2}-p_{3}^{2}) \end{array}$$

With the semi-classical Hamiltonian, the above-mentioned can further be used to obtain the dynamics potential and, in this way, we can study the dynamic nature of the HOBr’s highly excited vibrational states.

## 3. The Dynamics Potential of Highly Excited Vibration of HOBr

The dynamical potential of *H* (*q_i_*, *p_i_*, *P*) is the effective environment in which the *q_i_* coordinate stays for each P in a certain molecule. This is achieved by calculating the maximal and minimal energies by varying *p_i_* for each *q_i_* under the condition that n_2_ and n_3_ are nonnegative. The dynamical potential composed of these maximal and minimal energies as a function of *q_i_* is represented by a closed curve in which the quantal levels are enclosed. The dynamical potential also defines the *q_i_* region for each level it encloses. Furthermore, the points in the dynamical potential corresponding to ∂*H* ∂*q_i_=* 0 are fixed points in the dynamical space [5]. In this section, two main parts will be addressed as follows: (1) the dynamical potential influences of the H–O stretching model on the H–O–B bending model and the O–Br stretching model and (2) for instance, the phase space trajectories, the action integrals, and quantal environments of all energy levels in the dynamical potentials when *P* is equal to 27 will be studied.

### 3.1. The Study of Dynamical Potentials and Their Characteristics of Different Quantum Numbers *n*_1_ Corresponding to Typical Polyad Numbers in HOBr

**Figure 1:**
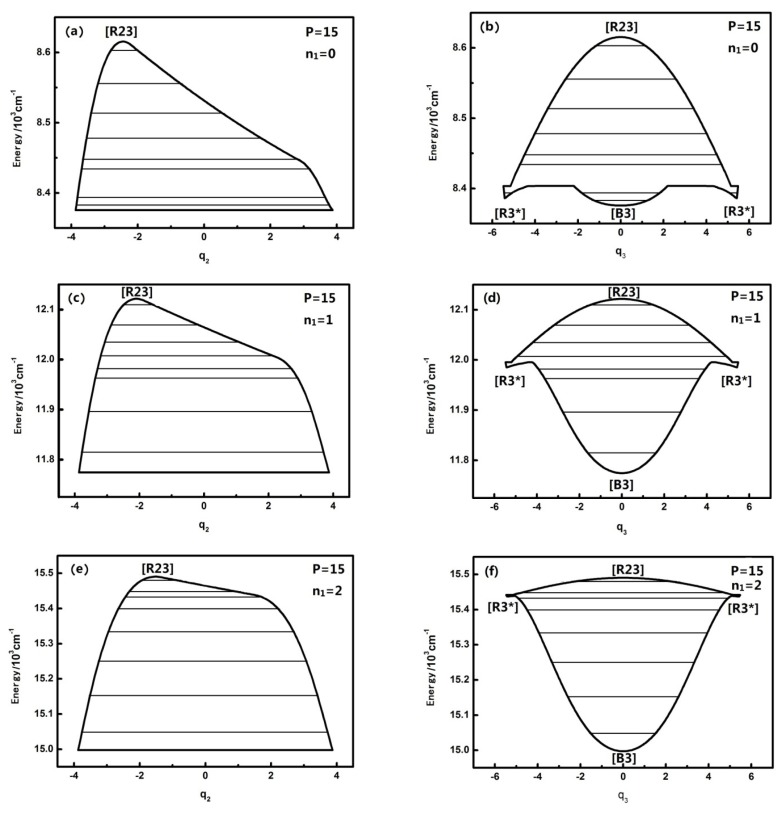

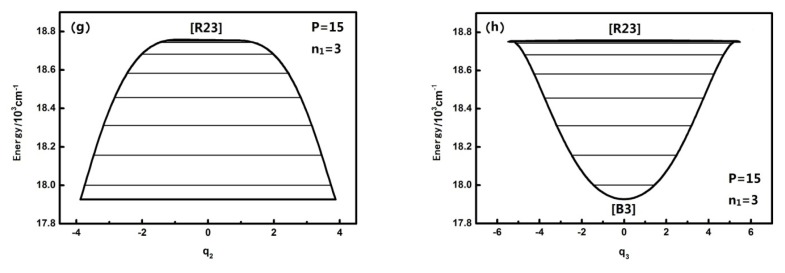
The dynamical potentials of HOBr (*P* = 15) with *n*_1_ = 0,1,2,3. The energy levels included in the P number are represented by the lines.

The dynamical potentials of HOBr (in coordinates *q*_2_, *q*_3_,) with *n*_1_ = 0,1,2,3 of different P numbers is considered, and their levels can be calculated with the aforementioned Hamiltonian (ground state from the potential energy at the bottom of 2764.0 cm^−1^). It is found that the dynamical potentials are different for small and large *P* numbers. The dynamical potential is shown as follows (the path marked by the fixed point is in accordance with the literature [13]) when *P* is equal to 15:

Figure 1 shows that when *n*_1_ is equal to 0,1,2,3, the dynamical potentials in the *q*_2_ coordinates are all simple inverse Morse potentials, which shows that the n_1_ mode has little effect on the dynamical potentials of the *q*_2_ coordinates. In the theory of the dynamical potential [9] it is noted that, for an inverse Morse potential, the stability of the lowest energy level is the worst in the inverted Morse potential corresponding to a certain *P*, which indicates that H–O stretching has no influence on the stability of the energy level corresponding to the H–O–Br bending vibration mode under the same *P* number. In the sense of the geometrical shape of the dynamical potential, it is found that the top of dynamical potentials only gradually flattens with the increase of n_1_, which shows that the n_1_ mode has almost no significant effect on the coupling of *n*_2_ and *n*_3_. In contrast, the dynamical potentials of HOBr in the *q*_3_ coordinate consist of an inverted Morse potential and a positive Morse potential, and with the increase of *n*_1_, the top of the inverted Morse potential gradually flattens and the positive Morse potential wells become deeper, which shows that the dynamical potential is turning into an almost pure positive Morse potential, especially when *n*_1_ is equal to three. As a result, the stability of the high energy levels becomes worse while the original fixed-point still remains the same. All the above phenomena show that, although the n_1_ mode is not coupled with the *n*_2_ and *n*_3_, it still has some effect on the resonant coupling of the other two modes, and this impact is reflected in the stability of the stretching mode (when *n*_1_ is equal to three, this impact is particularly evident). Or said differently, the H–O stretching mode has an important influence on the dynamics of the other degrees of freedom. According to qualitative analysis, this is because the intramolecular vibrational relaxation (IVR), caused by the resonance between *n*_2_ and *n*_3_, which enhances the stability of the energy levels [9], is weakened in the high-energy region. In the previous literature, the uncoupling modes in molecules have hardly ever been considered in the study of molecular dynamical behavior, and in this work, it is shown that the previous cognition maybe not comprehensive [2,3].

For larger *P* numbers, the change of the dynamical potentials is much more complicated. The dynamical potentials are shown as follows when *P* is, for instance, equal to 27:

When *n*_1_ is equal to three, the geometrical shape of the dynamical potentials of both *q*_2_ and *q*_3_ becomes increasingly complex and the number of the potential wells also increases. There are two inverted Morse in the *q*_2_ dynamical potentials, yet there are two positive Morse in the *q*_3_ dynamical potentials. This shows that with the increase of the *n*_1_ mode, the dynamical potential system consists of *n*_2_ and n_3_, which makes it more complex. This conclusion confirms once again that the n_1_ mode has some effect on the dynamical behavior of the other mutually coupled resonance modes and that this effect is more apparent compared with the case of *P* = 15, which shows that the uncoupling mode becomes much more important to the molecular dynamics behaviors with Polyad number.

**Figure 2:**
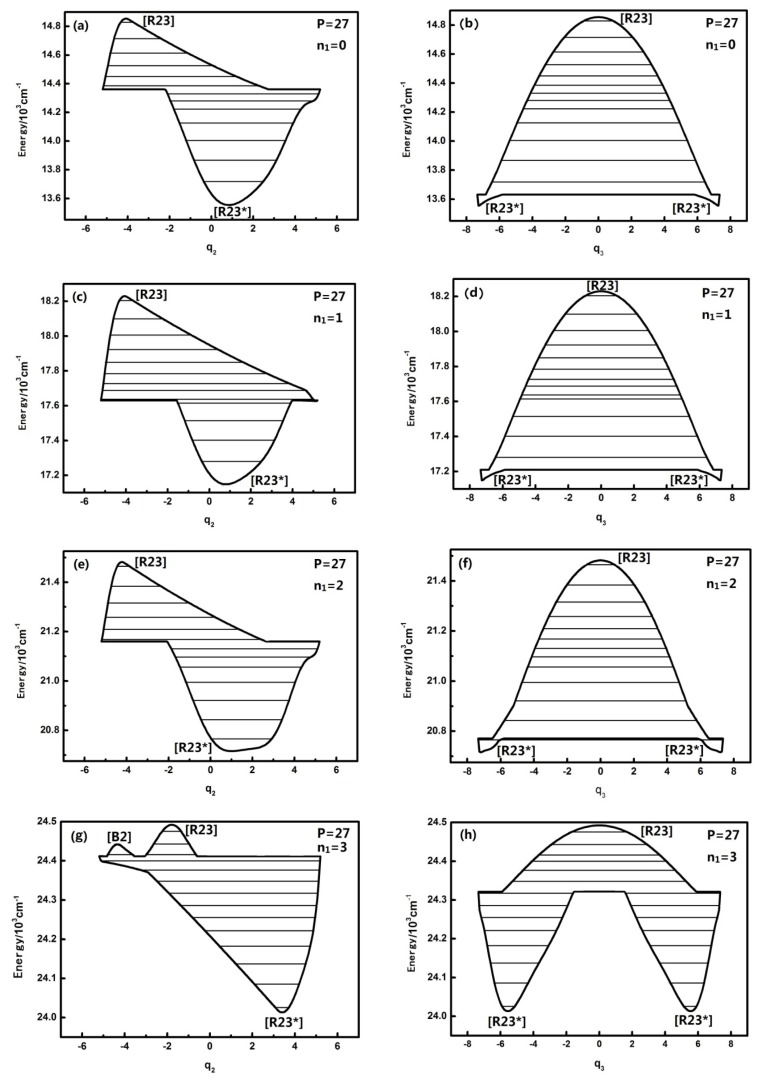
The dynamical potentials of HOBr (*P* = 27) with *n*_1_ = 0,1,2,3. The energy levels included in the P number are represented by the lines.

Figure 2 also shows that the *q*_2_ dynamical potential with *n*_1_ = 3 is more complex than the one with *n*_1_ = 0,1,2 and that the [*B*_2_] fixed point appears. This fixed point leads to the vibration modes corresponding to the highest three energy levels in the dynamical potential becoming localized vibrational when *n*_1_ is equal to three (in the two inverted Morse potentials). In addition, the fixed points of the dynamical potentials in *q*_3_ remain the same, but when *n*_1_ is equal to three, the vibration mode corresponding to the low energy level becomes localized vibrational (in the two positive Morse potentials). Compared with the dynamical potentials when P is equal to 15, it is obvious that the effect of n_1_ on *n*_2_ (or *n*_3_’s resonant coupling) is closely related to the value of the P number. The general conclusion is that n_1_ has little effect on the geometrical shape of the dynamical potential of *q*_2_, *q*_3_ when the P number is small, however, this effect becomes larger when the *P* number is larger.

In the above-mentioned study, it was found that although the H–O stretching mode was not coupled with the H–O–Br bending mode and the O–Br stretching mode, it still affected the resonant coupling of the two modes, thereby affecting the dynamics of HOBr. It is elucidated with qualitative analysis that the geometrical shape of the dynamical potentials and the corresponding fixed points are sensitive to the change of the H–O stretching mode in the sense of geometry. However, this phenomenon and the related explanation should be verified in further studies of other molecular systems.

### 3.2. The Nature and Quantal Environment of the Level of a Specific Polyad Number (*P* = 27)

**Figure 3:**
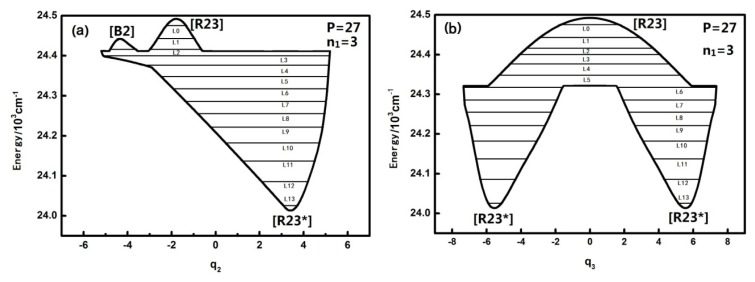
The dynamical potentials in *q*_2_ (**a**) and *q*_3_ (**b**) coordinates for HOBr with *P* = 27. The horizontal lines show the quantal states, L0–L13. [B], [R], [R*] are the stable and unstable fixed points, respectively.

The qualitatively different quantum number n_1_ of the HOBr system corresponding to dynamical potentials and characteristics are discussed in the previous section. Next we will discuss quantitatively the dynamical potentials of the specific *P* numbers with the analysis of the phase space trajectories and the action integral of every energy level. For instance, the dynamical potentials of HOBr with *P* = 27 are shown in Figure 3a,b in *q*_2_ and *q*_3_ coordinates, where the horizontal lines show the energy levels sharing the designated P. The reason why the case of *P* = 27 is singled out for discussion is that this case is quite representative and possesses the most fruitful characteristics. The dynamic potentials obtained when *n*_1_ is equal to three are as follows:

The energy is with respect to the ground state which is 2764.0 cm^−1^ above the bottom of the potential well, and the points in Figure 3 designated by [B], [R], [R*] are stable fixed points where both ∂*H*/∂*q_i_* and ∂*H*/∂*p_i_* (*i* = 2 or 3) are zero [10–13]. It is noted that [R23] is stable for the upper realm of the dynamical potential because the potential curve is inverse (upside down) and low energy regions have a positive potential at the bottom, which indicates that [R23*] is a stable fixed point. Similarly, in Figure 3a, [B2] is also a stable fixed point. Generally, dynamical potentials are considered to be able to find all of the fixed points by the two coordinates, and it is easy to find fixed points from intuitive geometry with the dynamics potential, which provides a convenient way to study the dynamics of a system.

**Figure 4:**
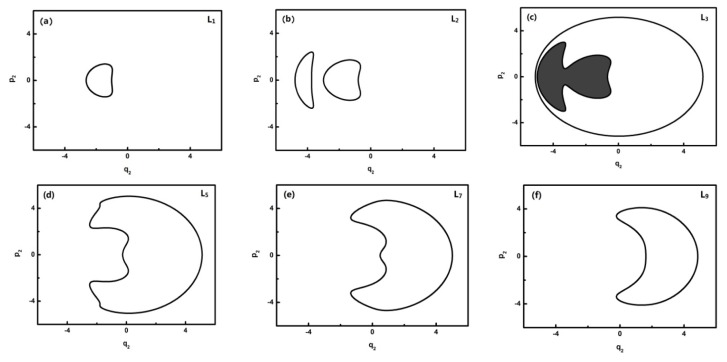
The trajectories of phase space of L1, L2, L3, L5, L7, L9 (*q*_2_ coordinates).

**Figure 5:**
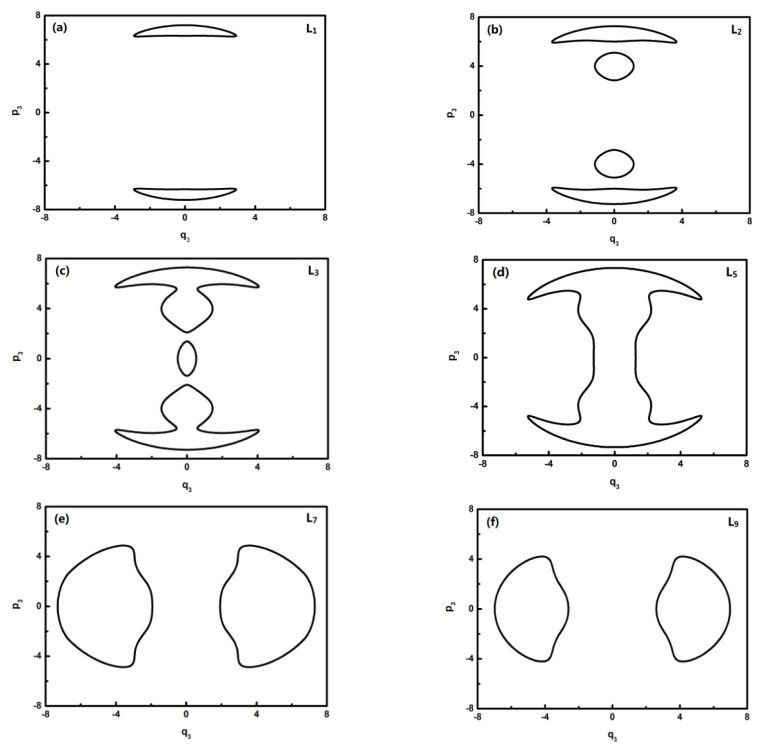
The trajectories of phase space of L1, L2, L3, L5, L7, L9 (*q*_3_ coordinates).

To further quantitatively analyze the features of related energy levels, the representative trajectories of phase space in *p_i_* − *q_i_* of each energy level is obtained. From the geometric properties of the pattern, the trajectory of phase space can be classified into different sets. It is found that the trajectories of L0–L1, L2, L3, L4–L8, L9–L13 in *q*_2_ coordinates constitute a class and the ones of the L0–L1, L2, L3, L4–L6, L7–L13 corresponding levels, respectively, constitute a class in *q*_3_ coordinates. They are partly shown in Figures 4 and 5:

In Figure 4, L0–L2 are in inverted Morse potentials and the area of the trajectory of the phase space increases with the reduction of energy. L3–L13 are in a positive Morse potential and the area of the trajectory of the phase space decreases with the reduction of energy. It is also found that the trajectory of the phase space of L2 is divided into two separate trajectories, of which L2 is located at the double-well potential in the dynamical potential shown in Figure 3.

In Figure 5, L0–L6 are in inverted Morse potentials and the area of the phase diagram increases with the reduction of energy. L7–L13 are in the positive Morse potential and the area of the phase diagram decreases with the reduction of energy. The trajectories of the phase space of L0–L1 are composed of two symmetrical parts (the upper one and lower one) because there is a positive and negative direction of momentum, and particularly, the trajectory of the phase space of L2 is composed of four symmetrical parts due to a multiplied effect of positive/negative directions of momentum on the energy level located in the double-well potential (Figure 3a). There is some difference in the phase space of L3, and it is shown that the trajectory is composed of three parts caused by an approximate harmonic vibration. Similarly, there are two parts of the trajectory in the phase space of L7–L13 because they are located in the two potential wells. Through the above-mentioned analysis, we found that the trajectories of the phase space can more intuitively reflect the dynamical characteristics of the vibration mode.

In order to study the quantal environment of each energy level, the calculation of the action integral of the phase space trajectories (quantum number) is needed and the corresponding formula is as follows:

(4) $$\text{Action integral}=\frac{1}{2\pi }\times \oint p_{i}dq_{i}$$

The results are shown in Table 2:

Table 2 shows the action integrals for the levels corresponding to *P* = 27, calculated from the trajectories of the phase space in (*q*_2_, *p*_2_) and (*q*_3_, *p*_3_). From the action integrals calculated from (*q*_2_, *p*_2_), the levels can be grouped into two subsets: L0 to L2 and L3 to L13. For the former ones, the action integral increases almost in a constant with the decline of energy levels due to the inverse-Morse well where they are located, and for the latter ones [15], the action integral decreases (also almost in a constant) with the decline of energy levels due to the positive Morse well where they are located. From the action integrals calculated from (*q*_3_, *p*_3_), the levels can be grouped into two subsets, also with constant increment (decrement) in their action integrals: L0 to L6 and L7 to L13. For the former ones, the action integral increases with the decline of energy levels due to the inverse-Morse well where they are located, but with minor deviation on L2, and for the latter ones, the action integral decreases with the decline of energy levels due to the positive Morse wells where they are located. The constant action integral increment/decrement demonstrates the compatibility of our classical treatment with the quantized levels and also shows that the levels stay in various dynamical environments, which are defined essentially by the classical fixed points though these energies belong to the same Polyad number. In addition, from the view of the (*q*_2_, *p*_2_) space, the difference between adjacent energy levels’ action integrals is almost always the same (approximately equal to one, but the L1 is an exception and should be further studied in the future). This value is equal to two if in the view of (*q*_2_, *p*_2_) because *P* = 2*n*_2_ + *n*_3_, which is also shown in Table 2. From the above results, it is elucidated that the action integral differential of adjacent energy levels nearly doubles that of (*q*_2_, *p*_2_), which corresponds exactly with the system of 2:1 Fermi resonance. The dynamics potential is actually the same as the trajectory connecting each fixed point, depicting the restrictive conditions in which individual energy levels are located.

## 4. Conclusions and Remarks

The highly excited vibrational state is hard to study using the quantum mechanical calculation because of its nonlinear interactions, but it is full of important information. The algebraic Hamiltonian, action integral and dynamical potential can bring direct physical pictures into the geometric sense of this research field. It is well known that the effective Hamiltonians with a single resonant interaction only are completely integrable systems and that the quantization can be done by the quantization of action integrals [16,17]. However, as an alternative method, the dynamical potential can lead to most inferences being drawn by the quantal wave function algorithm. The subtle behavior of the state wave functions that show a drastic change of mode character in a set of states sharing a common Polyad number is found to be related to the unstable region/fixed point in the dynamical space. This approach is global in the sense that the focus is on a set of levels instead of individual ones, which is different from traditional semi-classical methods, but, nevertheless, it should be noted that our approach is but one branch in the field of algebraic approaches to molecular vibrational dynamics. With the methods mentioned above using HOBr, it is not difficult to extend our subjects to other integrable and nonintegrable systems (such as O_3_, NO_2_, CS_2_, C_2_H_2_ and so on), including their dissociation, isomerization and dynamical symmetry. These conclusions are helpful and significant for us to understand the dynamics of molecular highly excited vibrational dynamics.

## Figures and Tables

**Table 1:** The coefficient of HOBr molecular vibration Hamiltonian.

Parameter name	Parameter values (cm^−1^)
*ω*_1_	3769.5381
*ω*_2_	1187.7106
*ω*_3_	622.3722
*x*_11_	−70.7877
*x*_12_	−26.8651
*x*_13_	10.3315
*x*_22_	−5.4432
*x*_23_	−3.7702
*x*_33_	−3.5507
*y_113_*	−3.4216
*y_122_*	−1.1955
*y_133_*	−0.9150
*y_223_*	−0.6071
*z_1133_*	0.2036
*z_1333_*	0.0121
*z_2333_*	−0.0015
*z_3333_*	−0.0006
*z_11112_*	0.3505
*z_11122_*	−0.6569
*z_11222_*	0.2578
*z_11223_*	0.0326
*z_12222_*	−0.0202
*z_22233_*	0.0033
*z_22333_*	−0.0017
*k_1_*	0.8717
*k_2_*	−0.3183
*k_3_*	−0.1935
*k_22_*	−0.0170

**Table 2:** The action integrals of the levels corresponding to *P* = 27, *n* = 3.

State label	Action integral in(*q*_2_, *p*_2_) space	Difference of the action integrals the neighboring levels	State label	Action integral in(*q*_3_, *p*_3_) space	Difference of the action integrals the neighboring levels
L0	0.20	/	L0	0.39	/
L1	0.61	0.41	L1	1.21	0.82
L2	1.64	1.03	L2	3.27	2.06
L3	10.83	/	L3	5.32	2.05
L4	9.45	1.38	L4	8.08	2.76
L5	8.29	1.14	L5	10.41	2.33
L6	7.18	1.11	L6	12.70	2.29
L7	6.12	1.06	L7	12.27	/
L8	5.13	0.99	L8	10.27	2.00
L9	4.19	0.94	L9	8.37	1.90
L10	3.19	1.00	L10	6.39	1.98
L11	2.19	1.00	L11	4.37	2.02
L12	1.18	1.01	L12	2.37	2.00
L13	0.19	0.99	L13	0.37	2.00
